# Structural and optical properties of penicillamine-protected gold nanocluster fractions separated by sequential size-selective fractionation

**DOI:** 10.3762/bjnano.10.96

**Published:** 2019-04-25

**Authors:** Xiupei Yang, Zhengli Yang, Fenglin Tang, Jing Xu, Maoxue Zhang, Martin M F Choi

**Affiliations:** 1 College of Chemistry and Chemical Engineering, China West Normal University, Nanchong 637000, China; 2 Partner State Key Laboratory of Environmental and Biological Analysis, and Department of Chemistry, Hong Kong Baptist University, 224 Waterloo Road, Kowloon Tong, Hong Kong SAR, China

**Keywords:** gold nanoclusters, monolayer-protected gold nanoclusters, sequential size-selective fractionation

## Abstract

Polydisperse water-soluble gold nanoclusters (AuNCs) protected by penicillamine have been synthesized in this work. The sequential size-selective precipitation (SSSP) technique has been applied for the size fractionation and purification of the monolayer-protected AuNCs. Through continuously adding acetone to a crude AuNC aqueous solution and controlling the volume percentage of acetone, we successfully separated the polydisperse AuNCs with diameters ranging from 0.5 to 5.4 nm into four different fractions sequentially. High-resolution transmission electron microscopy (HRTEM) shows that the four fractions are well-dispersed spherical particles of diameter 3.0 ± 0.6, 2.3 ± 0.5, 1.7 ± 0.4, and 1.2 ± 0.4 nm. Proton nuclear magnetic resonance spectroscopy suggests that disulfide, excess ligands and gold(I) complexes were removed from the AuNCs fractions. These results demonstrate the considerable potential of the SSSP technique for size-based separation and purification of AuNCs, achieving not only the isolation of larger nanoclusters (NCs) from small NCs in a continuous fashion, but also for the removal of small-molecule impurities. Based on the results from the mass spectrometry and thermogravimetric analysis, the average composition of the four fractions can be represented by Au_38_(SR)_18_, Au_28_(SR)_15_, Au_18_(SR)_12_, and Au_11_(SR)_8_, respectively. This indicates that the SSSP separation is mainly dependent on the core size and the ratio of Au atoms to ligands of AuNCs. X-ray photoelectron spectroscopy (XPS) has also been applied to observe the molecular dependence on the gold and sulfur chemical state of organosulfur monolayers of the fractions. The photoluminescence spectra of these AuNCs in the range of 900–790 nm was investigated at room temperature. The results show that the peak emission energy of the size-selected AuNCs undergoes a blue shift when the size is decreased, which can be attributed to the quantum confinement effect.

## Introduction

Due to the unique mechanical, chemical, thermal, magnetic and optical properties of nanometer-sized materials, they are often utilized in many fields [1]. Monolayer-protected gold nanoclusters (AuNCs) are nanoparticles of metal atoms stabilized by a protective layer of organic molecules [2]. These clusters are considered to be a hybrid system of small molecules and macroscopic materials, and are of great interest to the properties of gold core and organic monolayers [3]. The stable monolayer-protected NCs have enabled experiments to be carried out that would have been impossible or extremely difficult to conduct using less-stable materials. NCs can be handled like stable chemical compounds, which greatly facilitates their development of new applications and simplifies size dependence studies [4]. It is important to note that the preparation of high purity and monodisperse NCs is often critical because these variables can distort optical and electronic measurements, or hinder the self-assembly process of nanoscale structures [5]. Therefore, the effective control of the size distribution of NCs will facilitate the tunability of the properties of the nanomaterials, thereby providing opportunities for selective applications. Although many methods of AuNC synthesis with different surface functionalization have been developed [6], the products generally have a wide range of sizes (e.g., 2 to 12 nm) [7], and with some impurities [7–8]. Although the dispersion of the AuNCs can be improved to some extent by changing the ratio of the initial reactants (Au and ligand) and the synthesis conditions, or by other means such as heating [9], etching [10], and annealing [11], these methods are difficult to precisely control the size of the products. In addition, for the above mentioned methods, slight changes in reaction conditions may have a large effect on the product. Therefore, there are still major challenges in developing a strategy for synthesis of monodisperse NCs [12].

In order to obtain NCs with a low polydispersity index, various separation techniques have been proposed, including solvent fractionation [13], centrifugation [14], ultrafiltration [15], diafiltration [16], electrophoresis [17], and chromatography [18]. However, each of these separation technologies has its virtues as well as its limitations. Conventional solvent fractionation has the lowest separation resolution. Since the stationary phase used in size exclusion chromatography (SEC) has a high surface area, NCs with high surface activity can be irreversibly adsorbed by the column packing material, which limits its application to the separation of NCs. For capillary electrophoresis (CE) and high-performance liquid chromatography (HPLC), this limitation can be overcome to some extent by reducing the surface effects of the separation system. The capacity for each run of these two methods (micrograms for HPLC and nanograms for capillary zone electrophoresis (CZE)) have hindered them from widely being used for the fractionation of NCs into monodisperse sizes. Moreover, the fractionation of monolayer-protected NCs by other methods often depends on the core or ligand properties, in addition to being time-consuming and sophisticated. Size-selective precipitation is a simple and common method that has been used to the size fraction nanomaterial dispersions such as CdS and CdSe. However, this method has been mainly carried out to separate water-insoluble nanoparticles from organic solvents. On the other hand, it is also understood that the photophysical, electrochemical and other properties of AuNCs may be altered in the presence of excess thiol ligand; hence, it is necessary to develop a convenient, efficient, and stringent method for the purification of crude monolayer-protected NCs samples.

The purification of AuNCs is a very challenging task due to the similar solubility of water-soluble NCs and impurities. The separation by conventional purification techniques such as extraction, precipitation, chromatography or dialysis is incomplete. Some research in our group has already addressed the purification and separation of water-soluble *N*-acetyl-L-cysteine monolayer-protected nanoparticles (NPs). Although dialysis can be a useful means of removing impurities from NPs, it is a very time-consuming technique. Capillary electrophoresis provides an efficient and simple technique to fractionate water-soluble NCs [19], however it often depends on the specific core or ligand shell properties. At the same time, it is difficult to obtain similar size fractions in the separation process by conventional solvent fractionation. When HPLC and CE were used to separate NCs, the separation was complicated as many peaks were often observed in the polydisperse sample.

Recently, we have synthesized penicillamine-protected AuNCs in aqueous medium. The product was isolated and purified by sequential size-selective precipitation (SSSP) and characterized by mass spectrometry (MS) [20]. In order to further explore the different structures and properties of NCs of different sizes after fractionation, the crude AuNCs and fractions obtained from SSSP have been investigated by transmission electron microscopy (TEM), X-ray photoelectron spectroscopy (XPS), nuclear magnetic resonance (NMR), and UV–vis absorption together with photoluminescence (PL) spectroscopy.

## Experimental

### Chemicals and materials

D-Penicillamine (98%), chloroauric acid (HAuCl_4_·3H_2_O, 99.9%) and sodium borohydride (NaBH_4_, 99%) were purchased from Aldrich (Milwaukee, WI, USA). Acetone (99.5%), 2,5-dihydroxybenzoic acid (DHB, 99%), methanol (MeOH, 99.8%) and ethanol (EtOH, 99.8%) were obtained from Sigma (St. Louis, MO, USA). Acetic acid (HAc, 99.5%) and concentrated hydrochloric acid (37%) were obtained from Beijing Chemical Plant (Beijing, China). Deuterium oxide (D_2_O, 99.9%) was purchased from Fluka (Buchs, Switzerland). Purified water was used to prepare all solutions which was from a Milli-Q-RO_4_ water purification system (Millipore, Bedford, MA).

### Synthesis of gold nanoclusters

Penicillamine-protected AuNCs were synthesized using the procedures outlined in the following steps. First, 1.34 g of D-penicillamine was mixed with 1.18 g of HAuCl_4_·3H_2_O in 60 mL mixed solvent of MeOH/HAc (6:1 v/v) with stirring in ice bath. When the color of the solution changed to orange, the intermediate product was immediately reduced with 2.27 g NaBH_4_ in 6.0 mL of EtOH. Then 100 mL of acetone was added after 30 minutes, and the crude AuNCs were precipitated. Then, the crude AuNCs were redissolved in ≈0.2 mL of H_2_O, adjusted to a pH value to 1 by adding concentrated hydrochloric acid and then precipitated by acetone. Finally, the crude product was dried with nitrogen at 25 °C.

### Gold nanocluster fractionation

**Figure 1:**
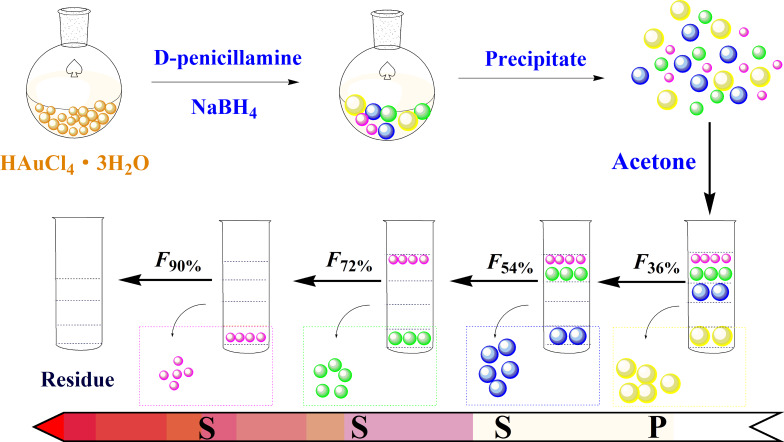
The scheme of the synthesis and fabrication of gold nanoclusters.

As can be seen from the TEM (vide infra) images, the initial product of the AuNCs was polydisperse. It is essential to narrow their size distribution in order to obtain their representative photophysical properties. As shown in Figure 1, SSSP was used to fractionation the crude AuNCs into four AuNCs fractions with different sizes. Briefly, organic solvent (acetone) was gradually added in an aqueous solution of AuNCs, whereby the AuNCs are gradually precipitated from large to small. By controlling the different proportions of acetone in the total volume, such as 36%, 54%, 72% and 90%, combined with centrifugation, we have obtained four AuNCs fractionations designated as *F*_36%_, *F*_54%_, *F*_72%_ and *F*_90%_, respectively. The remaining supernatant was dried with N_2_ which was designated as the residue.

### Characterization of the structure and optical properties of gold nanoclusters

High-resolution transmission electron microscopy (HRTEM) measurements were recorded on a JEOL 2010 transmission electron microscope (Tokyo, Japan) operating at an accelerating voltage of 200 kV. Proton nuclear magnetic resonance (^1^H NMR) spectra were performed on a Bruker AC 400 NMR spectrometer (Rheinstetten, Karlsruhe, Germany) in concentrated D_2_O solutions. At the same time, the fractions were investigated by a matrix-assisted laser desorption ionization time-of-flight mass spectrometer (MALDI-TOF MS) (Autoflex, Bruker, Germany). X-ray photoelectron spectroscopy (XPS) measurements were conducted with a Leybold Heraeus SKL-12 X-ray photoelectron spectrometer (Shenyang, China) modified with a VG CLAM 4 multichannel hemispherical analyzer using a Mg Kα excitation source at 1253.6 eV (10 kV, 20 mA). Thermogravimetric analysis (TGA) measurements were conducted with a Perkin-Elmer TGA 6 thermogravimetric analyzer (Waltham, MA, USA). In addition, the UV–vis absorption spectra of the crude AuNC product and the AuNC fractions were recorded on a Cary 100 Scan UV–vis spectrophotometer (Varian, Palo Alto, CA, USA). The PL properties of the samples were acquired on a QM4 spectrofluorometer (Photon Technology International, Lawrenceville, NJ, USA).

## Results and Discussion

### TEM characterization

**Figure 2:**
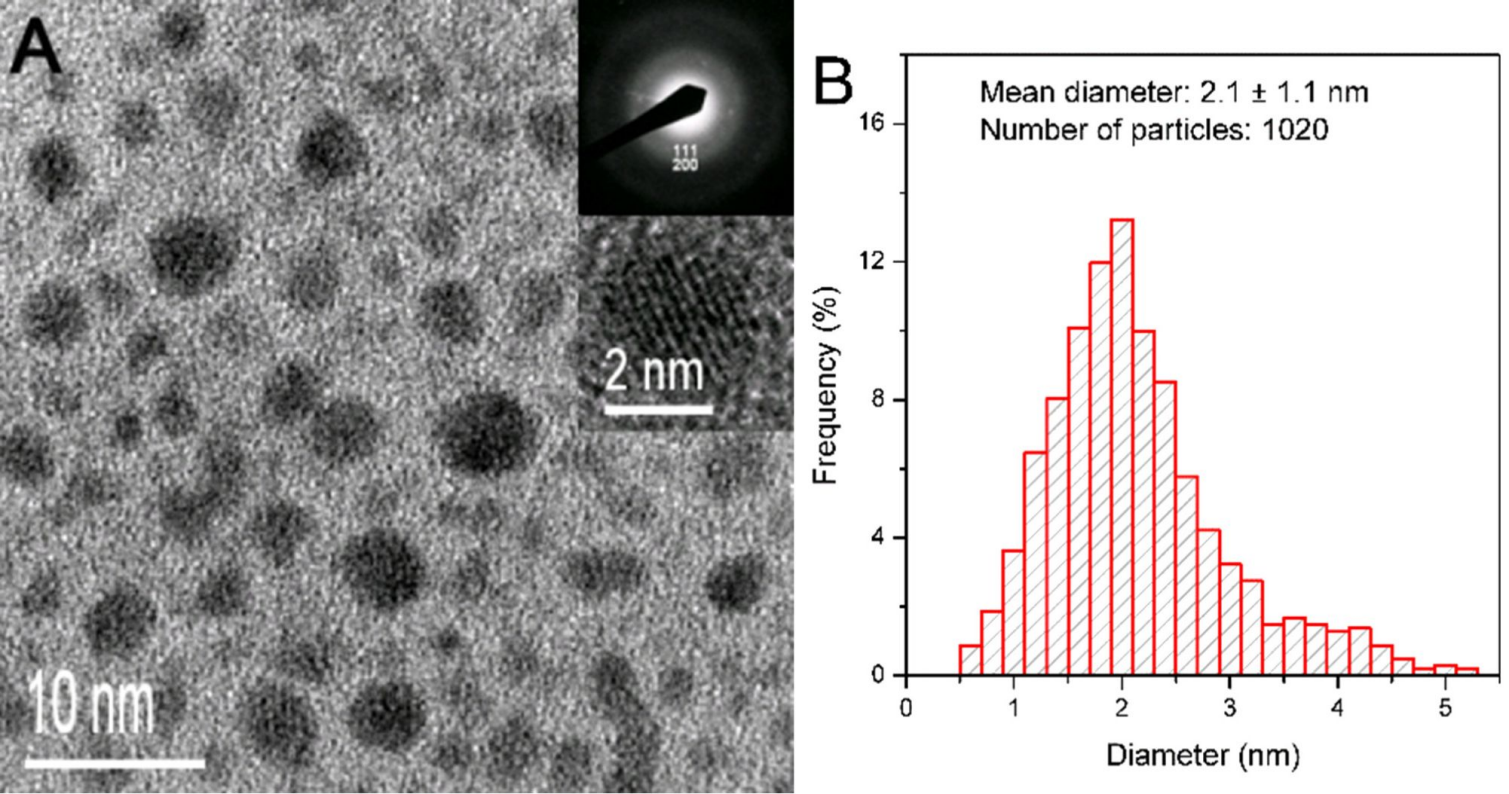
(A) HRTEM image of the crude penicillamin–AuNC product. The upper and lower insets display the SAED pattern and lattice fringe image of the AuNCs, respectively. (B) Size histogram of the crude penicillamine-protected AuNC product.

Figure 2A shows an HRTEM image of the crude AuNC product. It is clearly observed that the AuNC product is comprised of spherical particles of various sizes. The insets of Figure 2A show the selected area electron diffraction (SAED) pattern and high-magnification TEM image of a typical particle. The SAED pattern indicates the {111} and {200} planes of the typical face-centered cubic (fcc) structure of Au nanocrystal whereas the high-magnification TEM image also shows very clear fringes of the AuNC. These results infer that our AuNC product consists of highly crystalline nanocrystals. Figure 2B depicts the histogram of the size distribution obtained from the HRTEM image. The AuNC product exhibits a very broad particle size distribution, ranging from 0.5 to 5.4 nm, with an average core diameter of 2.1 ± 1.1 nm. Since the AuNC product is very polydisperse, the SSSP technique was employed to isolate the AuNC fractions with narrower size distribution from the AuNC product.

The size-selective precipitation technique can reduce the ability of a solvent to disperse clusters by adding a miscible non-solvent to the cluster agglomeration point. The larger clusters are first aggregated due to the greater van der Waals attraction between the clusters, and the clusters aggregated can be precipitated by centrifugation, and the smaller clusters are still dispersed in the solvent. Herein, we performed SSSP for the polydisperse penicillamine-coated water-soluble AuNCs from aqueous medium using acetone as a nonsolvent. When acetone was gradually added to the aqueous solution of AuNCs to achieve supersaturation of the solution, the solubility of AuNCs in the mixed solvent was lowered due to the low dielectric constant of acetone, resulting in the precipitation of relatively large-sized clusters. The deposit could then be separated from the solution by centrifugation. Four fractions (*F*_36%_, *F*_54%_, *F*_72%_, and *F*_90%_) from large to small have been obtained by repeating the above procedure and controlling the volume percentage of acetone corresponding to 36%, 54%, 72%, and 90%, respectively. The remaining soluble fraction is represented as *F*_0%_.

**Figure 3:**
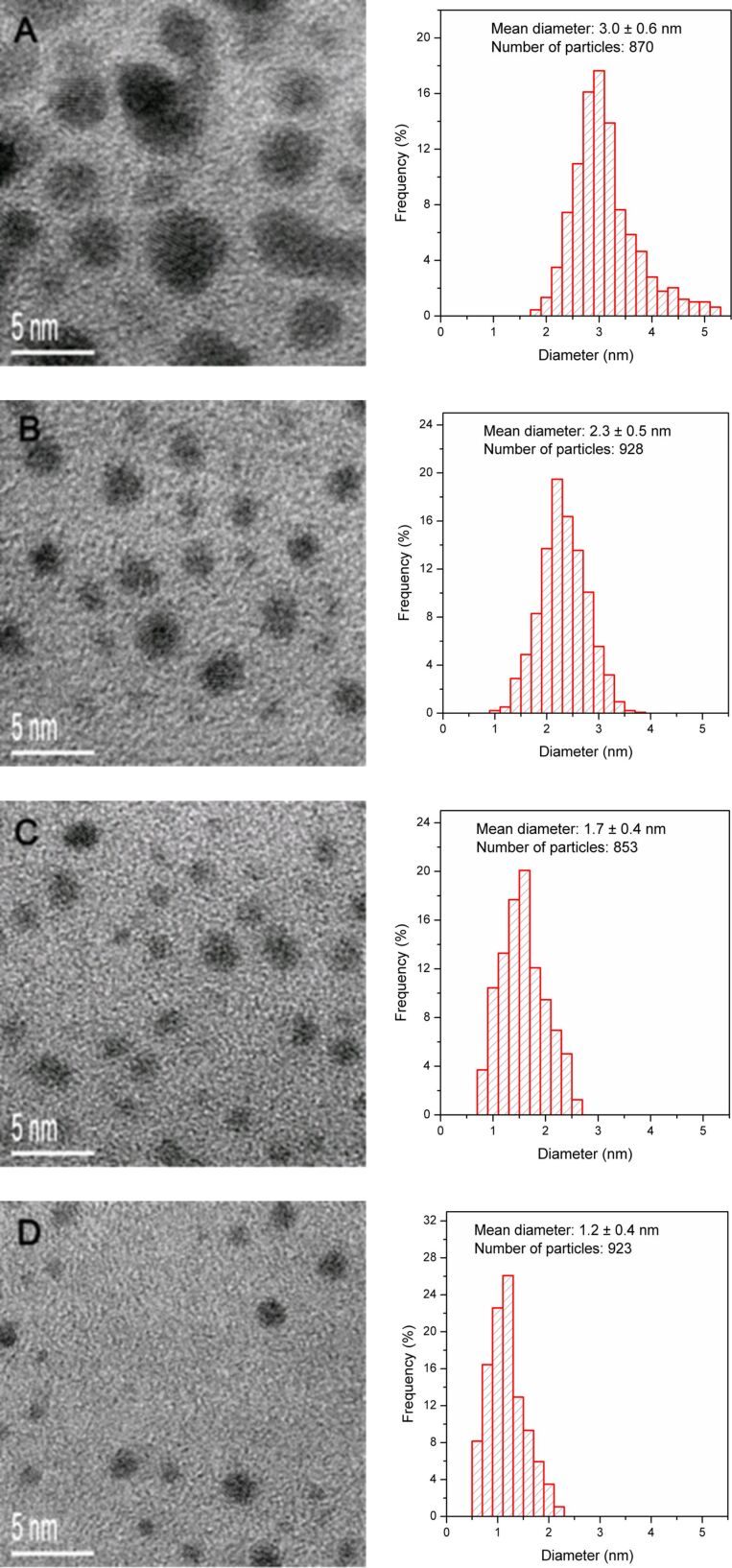
HRTEM images and size histograms of the sequential size-selected fractions (A) *F*_36%_, (B) *F*_54%_, (C) *F*_72%_, and (D) *F*_90%_.

Figure 3 displays the TEM images of the four AuNCs fractions (*F*_36%_, *F*_54%_, *F*_72%_, and *F*_90%_). Due to the fractionation process, these AuNCs are monodisperse relative to the crude product. The AuNC image was analyzed and a histogram was prepared for all AuNCs fractions, as shown in the right panel of Figure 3. The average cluster size is 3.0 ± 0.6, 2.3 ± 0.5, 1.7 ± 0.4 and 1.2 ± 0.4 nm for the four fractions, respectively. Obviously, these fractions show a decrease in both average size and size distribution compared to that observed before the size separation process (Figure 2). The fractionation process can improve the quality of the AuNCs by increasing the percentage of clusters at the median cluster size, especially for small AuNCs. For example, 6% of the crude product of AuNCs was 1.2 nm in diameter but after the fractionation process, 26% of the AuNCs in the fraction of *F*_72%_ were 1.2 nm. This indicates that the composition of the water/acetone mixed solvent greatly influences the precipitate morphology. It is demonstrated that the penicillamine-protected AuNCs of different average sizes can be separated from the aqueous solution dispersion by using the simple and effective SSSP technique. The standard deviation of the particle size decreases from 0.5 to 0.3 nm with an increase in the volume fraction of acetone in the AuNCs. This illustrates that a smaller change of volume percentage of acetone gives a more precise cluster separation due to a subtler change in solvent strength, leading to the isolation of the desired clusters of a specific size. However, it is quite difficult to obtain similar size fractions in the separation process. This issue can be addressed by accurately and carefully controlling the water/acetone proportion so that the required size fraction may be achieved.

### Selected-area electron diffraction characteristics of AuNCs fractions

**Figure 4:**
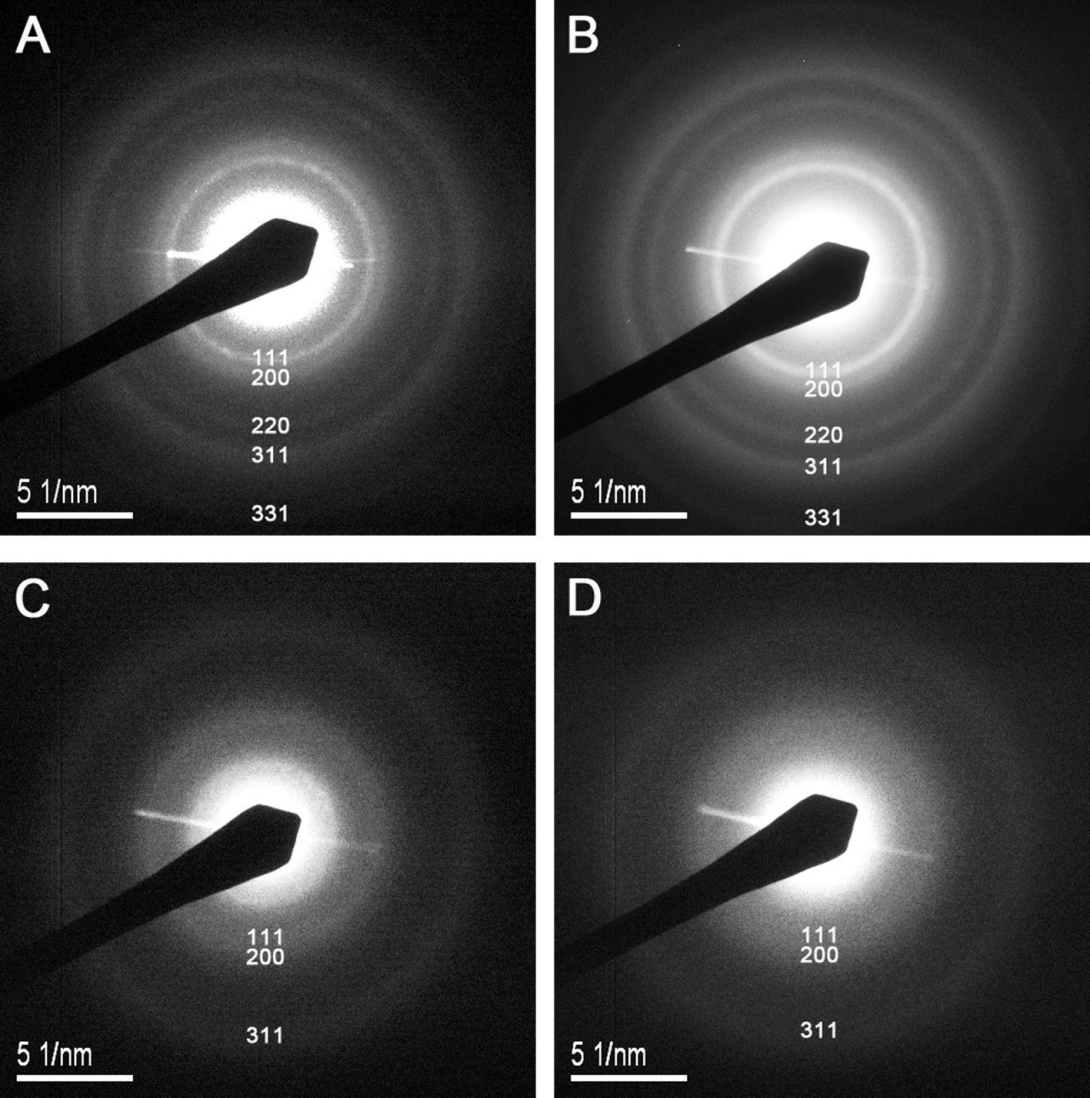
SAED patterns of the sequential size-selected fractions (A) *F*_36%_, (B) *F*_54%_, (C) *F*_7_*_2_*_%_, and (D) *F*_90%_.

The corresponding SAED pattern of each AuNC fraction was obtained by focusing the electron beam perpendicular to the TEM sample lying flat on the support carbon film. The blurry characteristic rings from inner to outer in the polycrystalline diffraction pattern can be indexed as {111}, {200}, {220}, {311}, and {331} planes of fcc Au crystals, where {*hkl*} are the Miller indices of the planes. The measured interplanar spacing along the growth direction of our AuNCs is 0.232 nm, corresponding well with the spacing between {111} lattice planes of the fcc Au crystals (0.235 nm). Figure 4 shows the individual SAED patterns of the four fractions (*F*_36%_, *F*_54%_, *F*_72%_, and *F*_90%_) obtained by SSSP. For the fractions *F*_36%_ and *F*_54%_, five diffraction rings with different radii and one center can be clearly observed. The structural characteristic of these AuNCs indicates the existence of an fcc structure of Au in the NCs and that the AuNCs have an anisotropic crystalline structure. This result is similar to that obtained by Lakshminarayanan for the diffraction characteristic of 1–3 nm thiol-monolayer-protected clusters [21]. As for the fractions *F*_72%_ and *F*_90%_, the {111} and {200} planes appear blurrier and the {220}, {311}, and {331} planes are not distinct from each other. Such observations may be due to the fact that the particle sizes of the latter two fractions *F*_72%_ and *F*_90%_ are smaller than those of the former two fractions *F*_36%_ and *F*_54%_. The diffraction image becomes gradually blurry with the decrease in the AuNC size. In addition, the diffraction rings broaden gradually with the reduction in particle size. A case in point is that the particle size of the fraction *F*_90%_ is so small (≈1.2 nm) that the rings are not discernable in the image and the corresponding diffraction ring is very broad compared to other fractions, indicating that these AuNCs are of polycrystalline structure.

### ^1^H NMR spectroscopy

**Figure 5:**
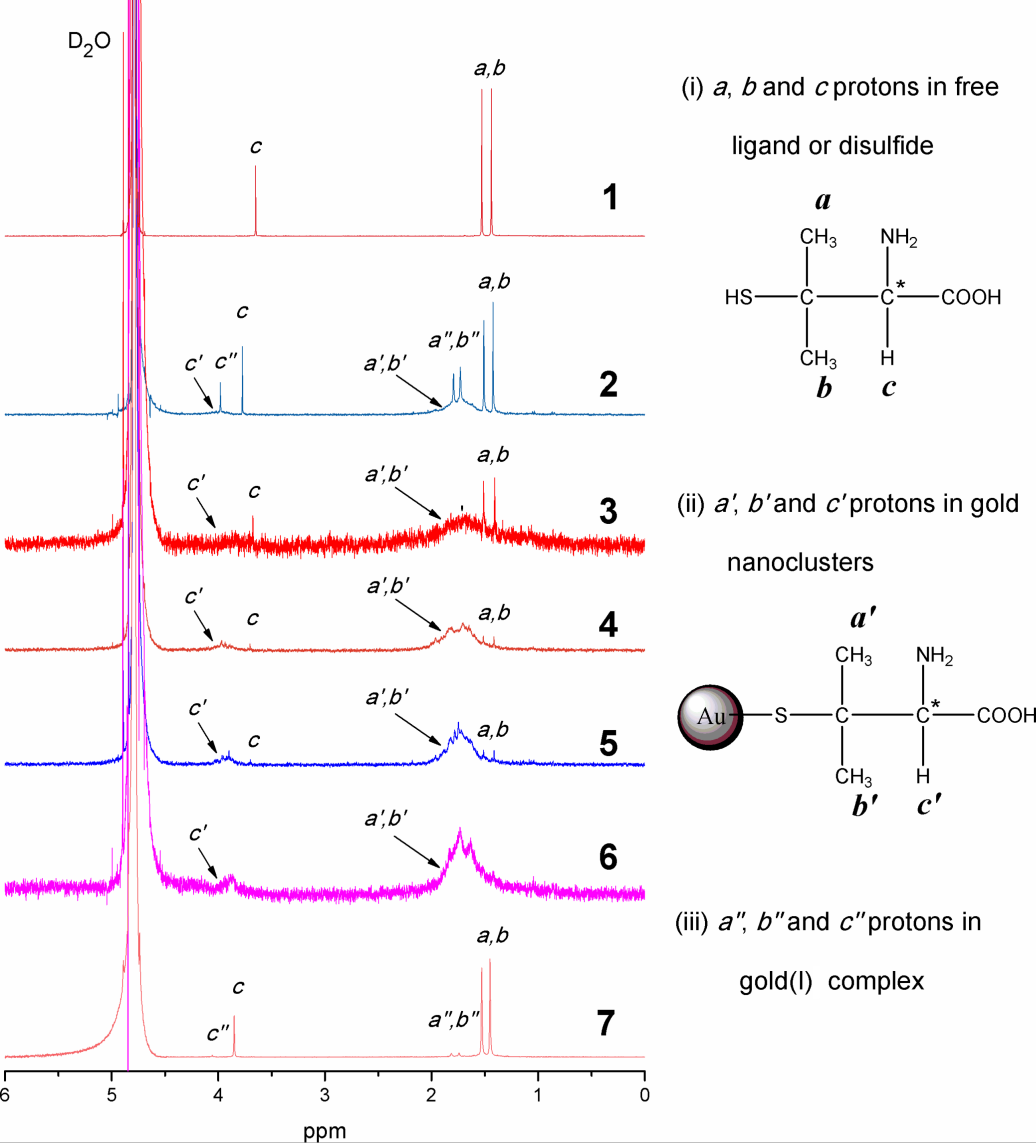
^1^H NMR spectra of the (1) free ligand penicillamine, (2) crude penicillamine-protected AuNC product and the sequential size-selected fractions (3) *F*_36%_, (4) *F*_54%_, (5) *F*_72%_, (6) *F*_90%_, and (7) *F*_0%_.

The spectra of D_2_O solutions of free ligand, crude and fractions obtained from SSSP are presented in Figure 5. The proton peaks are in the range of chemical shifts (δ) 1.4–4.8 ppm and the strong peak at 4.79 ppm is due to the residual H_2_O of the sample and HDO in D_2_O solvent. For ease of interpretation, the methyl proton (–CH_3_) peaks in the free penicillamine, AuNCs of the crude product and fractions *F*_36%_, *F*_54%_, *F*_72%_, *F*_90%_ and *F*_0%_, and byproducts penicillamine disulfide and gold(I) penicillamine complex are denoted as *a*, *b*, *a'*, *b'*, *a''* and *b''* whereas their corresponding methine proton (–CH–) peaks are labeled as *c*, *c'* and *c''* (right panel in Figure 5). In order to obtain a clear assignment, the assignment of the free ligand penicillamine has been investigated, and then this chemical shift information is used to help the peak assignment of AuNCs. The strong singlets at 1.44, 1.53 and 3.65 ppm (Figure 5, spectrum 1) are readily assigned to the methyl and methine protons of the free penicillamine as they do not couple with other protons. The methyl protons, which are labeled as *a* and *b* and appeared at 1.44 and 1.53 ppm, respectively, are chemically different in the ^1^H NMR spectrum due to the chirality effect [22] of the adjacent carbon (C* in the right panel of Figure 5). Peak splitting is also observed due to chiral induction in the ^1^H NMR spectrum of the crude product (spectrum 2) and size-selective fractions (Figure 5, spectra 3–6): the methyl protons are split into two peaks at ≈1.42 and 1.51 ppm, respectively.

It is tricky to assign the proton peaks of methyl and methine (Figure 5, spectrum 2) based on the chemical shifts of free penicillamine ligand. It becomes quite complicated due to three group peaks (labeled as *a b c*, *a' b' c'* and *a'' b'' c''*) observed. The sharp proton peaks at 1.42, 1.51, and 3.73 ppm (labeled as *a b c*) can be readily assigned to the methyl and methine protons in penicllamine and disulfide. The figure also shows that the spectrum of the crude exhibits two characteristic broadening peaks from 1.58 to 1.88 and from 3.88 to 4.10 ppm, which displays a slight chemical shift consistent with expectations for the presence of nanocluster-bound ligand [5]. This resonance broadening (relative to the monomer ligand) is due to the discontinuity of the diamagnetic susceptibility of the gold–hydrocarbon interface and the residual dipole interaction in the layer due to space constraints and confirms that penicillamine is bound to the gold particle surface [23]. Note that three sharp peaks residing on the broad peaks at δ 1.79, 3.77 and 3.98 ppm are possibly from residual gold(I) complex in our crude product. On the other hand, since the methyl and methine protons of the AuNC or gold(I) complex are both very close to the Au core, these proton signals are likely to be significantly downfield shifted.

The NMR spectra of fractions (Figure 5, spectra 3–6) obtained by SSSP show significantly reduced sharp signals, indicating that materials not coupled to the NCs (free ligand, disulfide and gold(I) complex) [24] have been almost completely removed in the SSSP steps. In addition, as expected [25], the broadening increased the AuNC core size (from fraction *F*_90%_ to fraction *F*_36%_). This is qualitatively consistent with rotational spin relaxation (*T*_2_); that is, the larger 3.0 nm average core size AuNCs (Figure 5, spectrum 3) are significantly slower than the smallest 1.2 nm average core size (Figure 5, spectrum 6) [26]. Further expansion of the packing density gradient along the ligand chain may result in a broader methyl resonance (δ = 1.4–1.9) than a methine proton (δ 3.7–4.0) located farther away from the core by the effects of steric hindrance. It is noteworthy that the spectrum of the sample dried from the rest soluble fraction (*F*_0%_) preserves the characteristic chemical shift at 1.42, 1.51, 1.73, 1.79, 3.77 and 3.98 ppm (Figure 5, spectrum 7), indicating that the main disulfide with a small amount of free ligand and gold(I) complex are separated from other fractions. The assignments are also confirmed by mass spectrometry (vide infra). That is to say that not only is the size distribution of the AuNCs narrowed, but also the excess free ligand, disulfide and gold(I) complex, can be removed.

### Mass spectrometry and thermogravimetric studies of SSSP fractions

**Figure 6:**
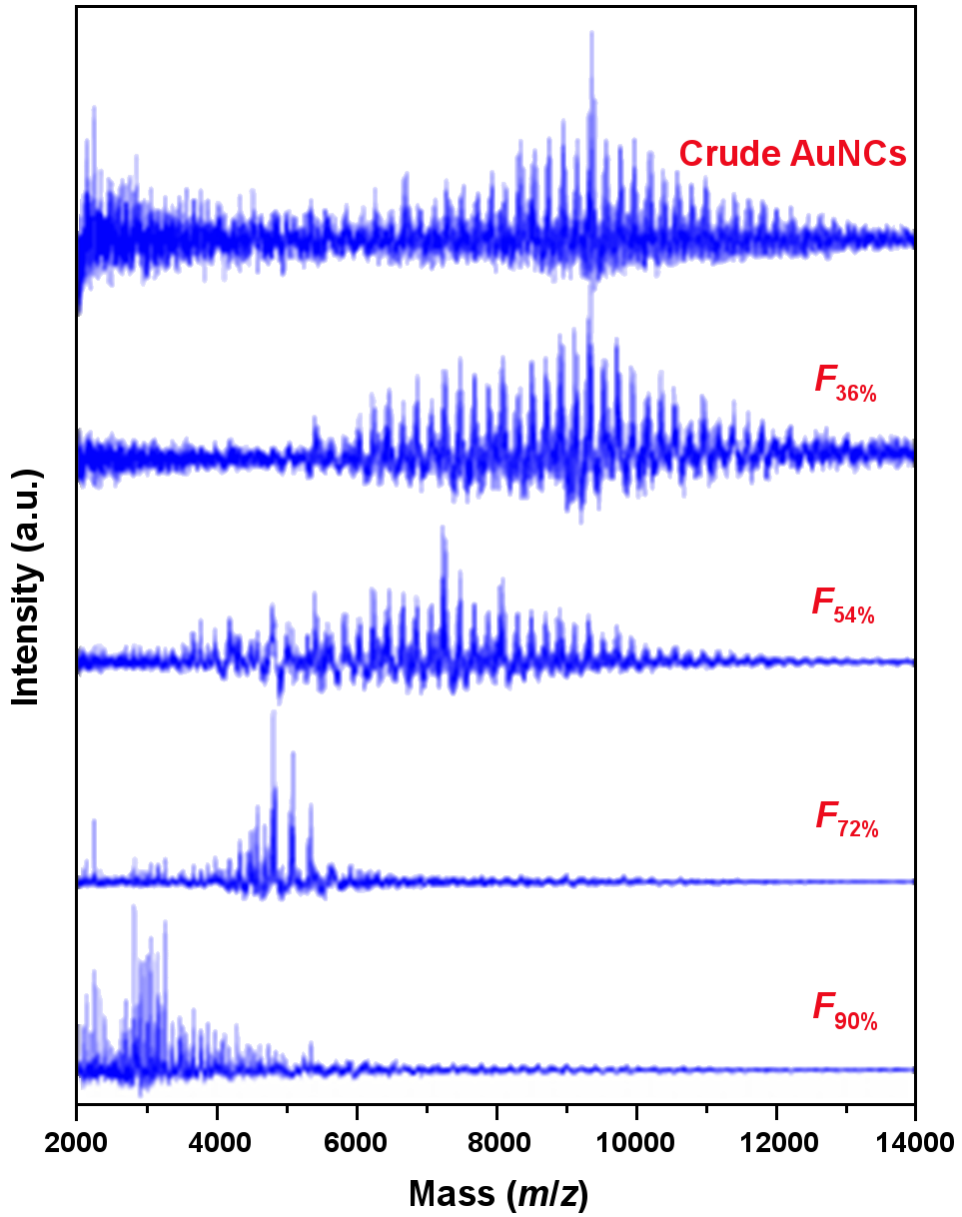
MALDI-TOF mass spectra of the crude AuNC product and SSSP fractions after baseline subtraction.

Matrix-assisted laser desorption/ionization time-of-flight mass spectrometry (MALDI–MS) is an effective method for determining the core size of NPs and has been used to track the fractional precipitation process of C12-NCs [27–28]. As such, MALDI–MS was applied to characterize the crude penicillamine-protected AuNCs and as-obtained four fractions (Figure 6). In addition, the thermogravimetric analysis (TGA) of the AuNC samples was carried out and the weight loss of the crude AuNCs product, *F*_36%_, *F*_54%_, *F*_72%_, and *F*_90%_ fractions were calculated as well*.* Based on the TGA and MS results, the average molecular formula of the four fractions could be determined as Au_38_(SR)_18_, Au_28_(SR)_15_, Au_18_(SR)_12_, and Au_11_(SR)_8_, respectively. The corresponding results are consistent with our previous report [20]. However, the compositions proposed here are different from those well accepted, such as Au_38_(SR)_24_, Au_25_(SR)_18_, and Au_18_(SR)_14_. The discrepancy may be due to the amino group in the penicillamine molecule. We conclude that the AuNCs here are primarily protected by the Au–S bonds. However, we cannot rule out that a small amount of Au–N bonds are present in the clusters.

### X-ray photoelectron analysis

**Figure 7:**
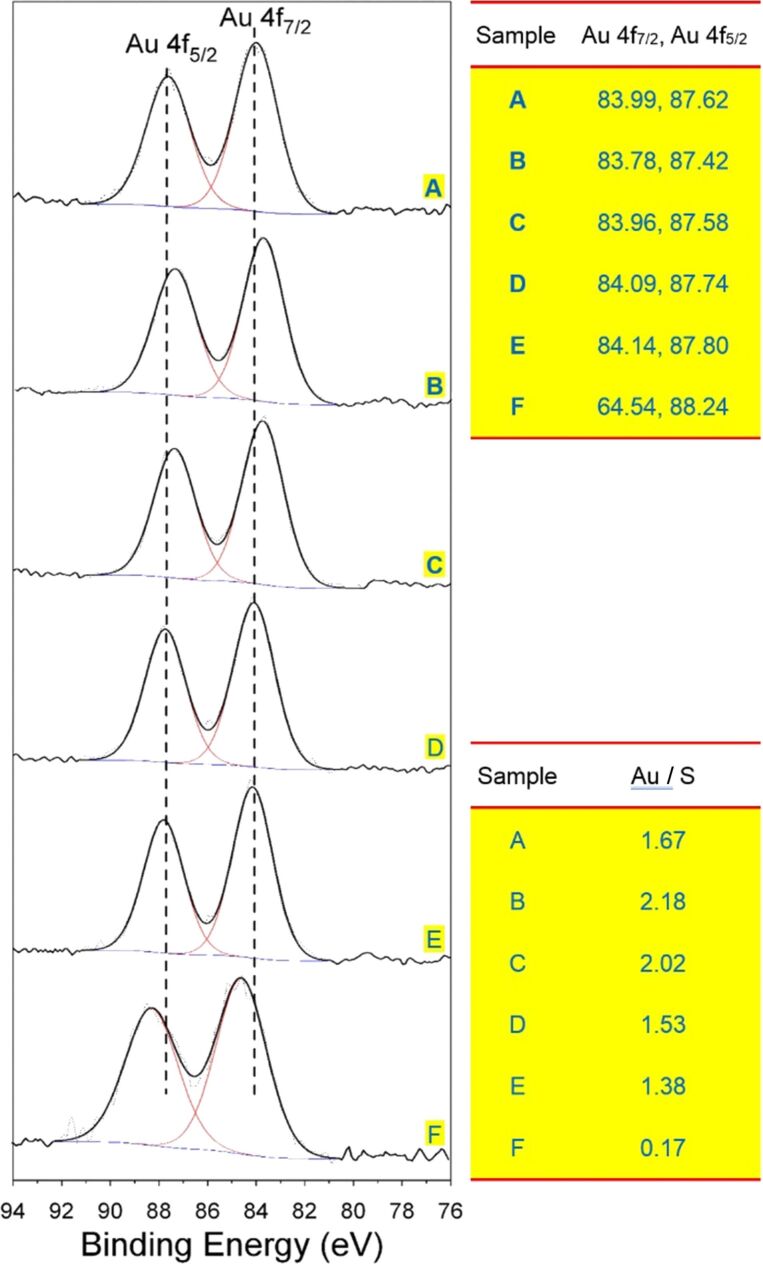
XPS spectra in the Au 4f region of the (A) crude penicillamine-protected AuNC product and the sequential size-selected fractions (B) *F*_36%_, (C) *F*_54%_, (D) *F*_72%_, (E) *F*_90%_, and (F) *F*_0%_. The right panels display the Au 4f_7/2_ and Au 4f_5/2_ binding energies and Au/S ratio of the samples determined by XPS.

**Figure 8:**
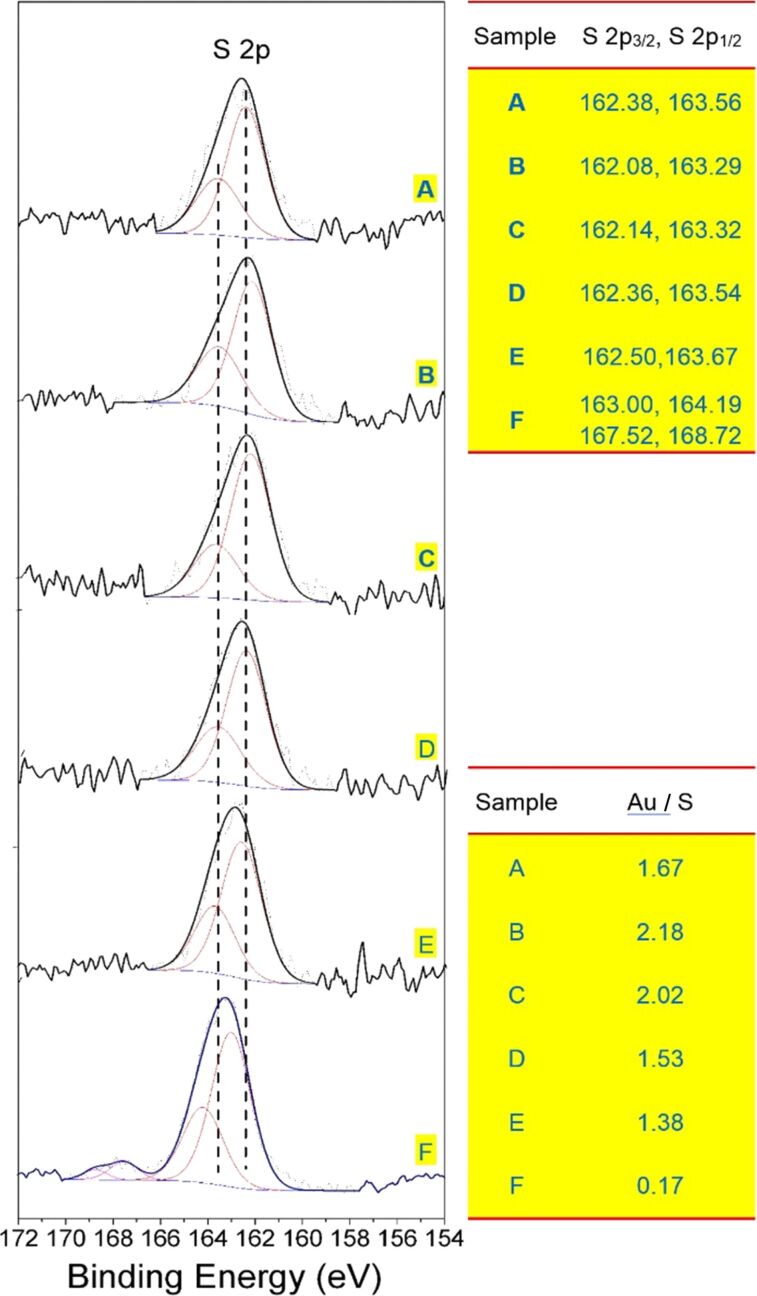
XPS spectra in the S 2p region of the (A) crude penicillamine-protected AuNC product and the sequential size-selected fractions (B) *F_36%_*, (C) *F_54%_*, (D) *F_72%_*, (E) *F_90%_*, and (F) *F_0%_*. The right panel displays the S 2p_3/2_ and S 2p_1/2_ binding energies of the samples.

XPS can be used not only to analyze the properties of S–Au bonds in gold nanoparticles, but also to analyze the atomic composition of samples [29]. In this study, we performed XPS analysis of the crude AuNC product and the SSSP fractions using the C 1S peak (284.6 eV) as a reference. Figure 7 presents the corresponding Au 4f spectra of the crude AuNC product and the fraction samples. Compared to the positions of the Au 4f peaks, as the size of the nanoclusters decreases, there is a transition to higher binding energy. This kinetic energy loss is the result of the temporary charge of the nanoclusters caused by the photoionization process [30]. Thus, the Au 4f peaks for the first fraction (*F*_36%_), also the largest fraction, appear at the lowest binding energy (Au 4f_7/2_ 83.78eV) among those fractions, whereas the same peaks for the smaller clusters approach the value previously observed for the Au(I) polymer (84.9 eV) [31]. The XPS spectra exhibit a Au 4f_7/2_ level with a binding energy of 83.96, 84.09 and 84.14 eV for the *F*_54%_, *F*_72%_, and *F*_90%_ fractions, respectively. This kind of shift in binding energy is very common for very small gold particles due to reduced core-hole screening in small metal particles. This indicates that there are significant differences in the electronic properties of very small particles and bulk materials, and that the dimensionally dependent changes in the electronic structure produce unusual characteristics [32]. The binding energies of the latter fraction are higher than those of the former fraction, further indicating that the gold cores of the latter were more positively charged than those of the former. Although there are only few reports of positively charged thiol-protected AuNCs, the chemical oxidation of [Au_25_(SC_6_H_13_)_18_]^0^ described in [33] yielded positively charged AuNCs. The Au 4f_7/2_ binding energy in the residue fraction (*F*_0%_), however, is shifted by 0.76 eV with respect to the largest clusters (*F*_36%_). The trend of this shift is closely related to the oxidation state of Au, and its magnitude is similar to that of other Au(I) complexes [31]. The analysis of the S 2p peak is a reliable method for assessing the chemical bonds of sulfur atoms in AuNCs. Figure 8 depicts the XPS spectra of the crude AuNC product and fractions of *F*_36%_, *F*_54%_, *F*_72%_, *F*_90%_ and *F*_0%_ in the S 2p region. Since the S 2p is a broad peak (solid line), it can be deconvoluted into two narrower peaks (dash lines) corresponding to a doublet at 162 eV (2p_3/2_) and 163.2 eV (2p_1/2_). Each doublet has a peak area ratio of 2:1 and a splitting of ≈1.2 eV that is consistent with the results determined theoretically by the spin–orbit splitting effect. The sulfur binding energy for S 2p_3/2_ at 162 eV suggests that all the sulfur atoms are bound to gold as a thiolate species [34]. Unbound thiols (164 eV) cannot be found in the XPS spectra of the five fractions nor can the oxidized sulfur species (S 2p > 166 eV) be observed [35]. The observation confirms the stability of the NCs. The sulfur S 2p_3/2_ binding energy values (Figure 8) are 162.08, 162.14, 162.36 and 162.50 eV for fractions *F*_36%_, *F*_54%_, *F*_72%_ and *F*_90%_, respectively. Considering the S 2p_3/2_ binding energy of the element S(S8) is 164.2 eV, this shift corresponds to the negatively charge S adsorbed on the metal [36]. For smaller sized clusters, there is a subtle shift (0.3–0.4 eV) to higher binding energy. The peaks of the smaller core size shift to a higher binding energy, indicating a change in the properties of the Au–S bond or an increase in the effect of the final state charge effect [37]. Note that the S 2p_3/2_ binding energy in the *F*_0%_ fraction is slightly more positive than that of the other fractions (163.00 eV). These shifts are due to free ligands, disulfides and gold(I) complexes in this fraction. Additionally, a weak shoulder peak at around 168 eV is observed as shown in Figure 8F, which is assignable to sulfur oxide generated from the fractionation procedure. Upon exposure to atmospheric oxygen, two thiol residues combine to form a disulfide bridge through a thiocarbonyl group, which can be further oxidized in air to form a thiosulfonate [34].

The Au/S atom ratios calculated from integrated Au 4f and S 2p peak intensities measured in high-resolution scans are also shown in the right bottom panel of Figure 7. The decrease in the Au/S atom ratio with the size of the nanocluster clearly shows that the smaller AuNCs possess higher surface coverage ratio of penicillamine. Actually, on the basis of the results, we can easily understand why the smaller NCs precipitate in the higher volume percent acetone (the smaller NCs are protected by relative more hydrophilic ligand). The Au/S ratio decreases with the decrease in the cluster core size which is also verified by the TGA results. TGA data showed that the percentage of ligands in AuNCs increased as the cluster size decreased.

A small amount of Na and Cl were found in the crude product by XPS analysis, indicating that traces of starting material were not completely removed during the synthesis of AuNCs. Fortunately, these impurities can be effectively eliminated with the SSSP procedure and no Na and Cl peaks are found in the SSSP fractions. On the other hand, a significant increase in the Na and Cl content is observed in the XPS spectrum of the fraction *F*_0%_ (XPS data not shown). Our XPS data further demonstrates that the SSSP technique not only can narrow the size distribution of the AuNCs but also aids in purifying AuNC samples.

### Optical absorption and photoluminescence properties

**Figure 9:**
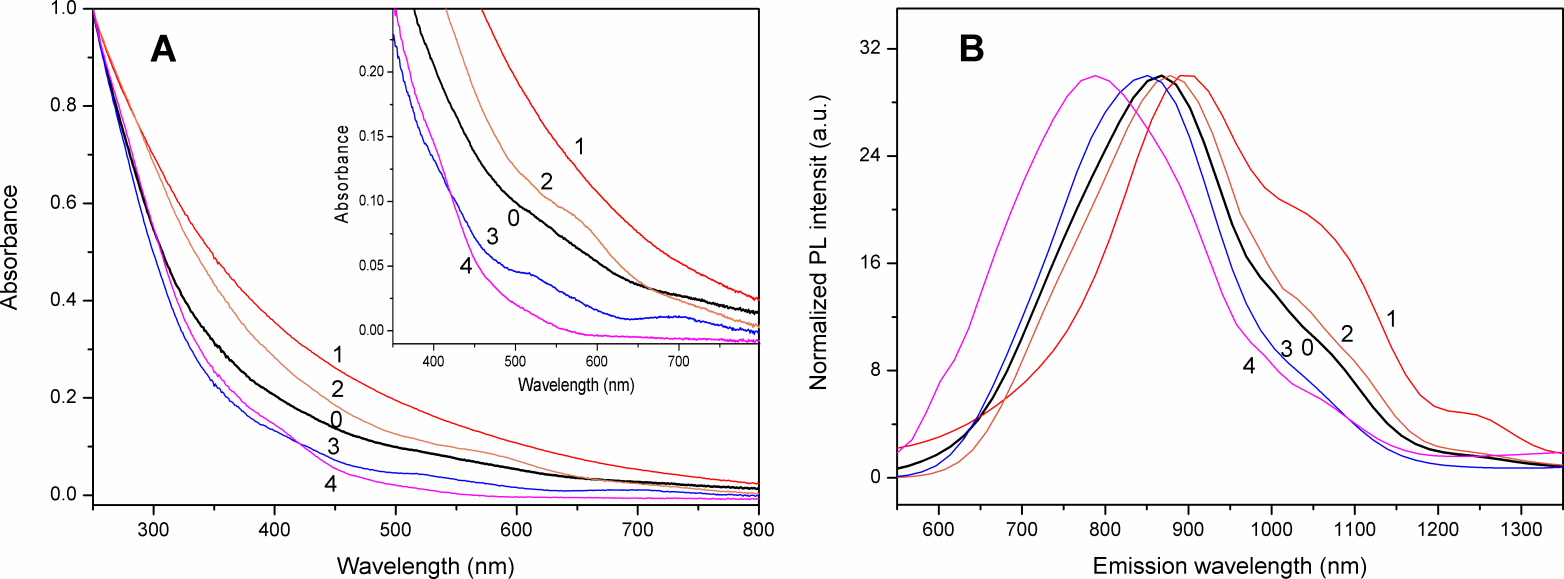
(A) UV–visible absorption and (B) corrected photoluminescence spectra (excited at 380 nm) of the (0) crude penicillamine-protected AuNC product and the sequential size-selected fractions (1) *F*_36%_, (2) *F*_54%_, (3) *F*_72%_, and (4) *F*_90%_. The UV–visible spectra are normalized at 250 nm to remove the effect on concentration difference. The inset depicts the enlarged UV–visible spectra in the range 350−800 nm for clarity and ease of comparison.

UV–vis absorption spectra of the AuNC crude product and the fractions are displayed in Figure 9. In order to eliminate the effect of concentration changes on the comparison of spectral shape and band position, these spectra are normalized at 250 nm. The smaller AuNCs possess a sharper decrease in absorbance than that of larger ones, from shorter to longer wavelengths, as could be observed in previous work [38–39]. In essence, the large AuNCs produce absorption spectra that have higher absorption than that of the small core nanoclusters. The difference in the absorption spectra of the four NCs fractions clearly demonstrates the change in core size and distribution of the NCs obtained from SSSP fractionation. The spectral characteristics of the small and large core size of AuNCs are in line with the previously reported AuNC spectra. When the core diameter of AuNC is smaller than 3 nm, the surface plasmon resonance band broadens into the baseline and the absorption spectra show only the characteristic exponential decay curve [40]. For even smaller AuNCs, some molecular features may begin to appear because of the presence of HOMO–LUMO band gaps [41]. The inset of Figure 9 indicates the UV–vis absorption spectra of the AuNC crude product and the fractions in the range 350–800 nm. There is the only a featureless absorption profile observed for the first precipitated fraction (*F*_36%_, Au_38_ clusters). In contrast, one well-defined absorption shoulder peak appears at 576 nm for the second precipitated fraction (*F*_54%_, Au_28_ clusters). For the third precipitated fraction (*F*_72%_, Au_18_ clusters), three well-defined absorption maximum and shoulder peaks appear about 417, 514, and 691 nm, resulting from the NC interband electronic transitions and the excitonic transition of the HOMO–LUMO bandgap of the subnanometer-sized NCs [42]. For the final precipitated fraction (*F*_90%_, Au_11_ clusters), its UV absorption decays to visible light in an approximately exponential manner with no detectable surface plasmon spectral bands.

All the normalized PL spectra for the crude and size-selective fraction samples in Figure 9B were obtained at 25 °C at an excitation wavelength of 380 nm. The maximum emission peak of the PL spectrum of the original sample is 867 nm, while the fluorescence emission from the first fraction to the last fraction obtained by SSSP shows a blue shift, that is, peaks of 900, 878, 845 and 790 nm, respectively. For size-dependent PL, the shorter PL peak positions are related to the smaller cluster sizes [43]. Figure 9B obviously indicates that tuning of the PL emission is possible by adjusting the volume percentage of acetone in the SSSP process. It is evident that AuNCs with an average size of 3.0 nm and above in the first precipitated fraction emit at a wavelength longer than 900 nm. When the cluster size decreases from 3.0 to less than 1.2 nm, the PL peak energy shifts toward shorter wavelengths from 900 to 790 nm. Such dependence of the PL peak energies on nanocluster sizes indicates that the optical luminescence of the samples comes from the quantum confinement effect. The band gap of the NCs increases with a decrease in the dimension of the AuNC quantum structure.

## Conclusion

In this research, a highly polydisperse penicillamine-protected AuNC product was initially synthesized in aqueous medium. We have demonstrated that SSSP is an effective, convenient and simple method for the purification and size-based separation of water-soluble monolayer-protected AuNCs. Four fractions of AuNCs with average Au_11_, Au_18_, Au_28_, and Au_38_ were achieved from the polydisperse product by SSSP. The structural and optical properties of the AuNC fractions were further investigated by TEM, ^1^H NMR, MS and XPS. Obtaining a narrow size distribution and higher purity AuNCs by SSSP will allow their optical, electronic and catalytic properties to be determined with high precision, facilitating easy evaluation of structure–function relationships in their toxicological studies, as well as improving the repeatability in AuNC self-assembly. Further optimization of SSSP fractionation can result in fractionation of AuNCs to specific sizes at gram scales, which is significant for expanding its specific application.
